# Analysis of the Correlation Between MDS Inflammatory Indicators and Clinical Characteristics: A Comprehensive Analysis

**DOI:** 10.1155/mi/8812329

**Published:** 2025-06-19

**Authors:** ZhongLi Hu, Lijia Pei, ZhongTing Hu, YanLi Yang, YuXian Wang, Mengqing Hua, Ping Zhao

**Affiliations:** ^1^Department of Haematology, The First Affiliated Hospital of Bengbu Medical University, Bengbu, Anhui, China; ^2^Anhui Province Key Laboratory of Immunology in Chronic Diseases, Bengbu Medical University, Bengbu, Anhui, China; ^3^Department of Orthopaedics, The First Affiliated Hospital of Bengbu Medical University, Bengbu, Anhui, China; ^4^Office of Academic Research, Bengbu Medical University, Bengbu, Anhui, China; ^5^Department of Rheumatology, The First Affiliated Hospital of Bengbu Medical University, Bengbu, Anhui, China

**Keywords:** CD34, inflammatory markers, monocyte/lymphocyte ratio, myelodysplastic syndrome, neutrophil/lymphocyte ratio, platelet/lymphocyte ratio

## Abstract

**Objective:** This study aims to investigate the correlations among the neutrophil-to-lymphocyte ratio (NLR), monocyte-to-lymphocyte ratio (MLR), and platelet-to-lymphocyte ratio (PLR) at the initial diagnosis of myelodysplastic syndrome (MDS) and the clinical characteristics of patients. It explores the correlation between MDS and inflammatory markers, providing a basis for early assessment of treatment efficacy, diagnosis, and guiding personalized treatment.

**Methods:** Analyzing the bone marrow smears and flow cytometry immunophenotyping of 70 MDS patients. Conducting a comparative analysis of the multi-parameter linear correlation between the clinical characteristics of MDS patients and inflammatory markers, comparing the differences in inflammatory markers between MDS patients and the control group. Additionally, investigating the differential correlation analysis between different primitive cell ratios, different MDS types, different immunophenotypic characteristics, different morphological features, and inflammatory markers.

**Results:** Comparative analysis between MDS and the control group revealed that NLR and PLR have good diagnostic specificity (area under the curve [AUC] = 0.9169, 0.8312). When comparing the clinical characteristics of MDS patients, MLR showed a strong correlation with the total white blood cell count at the initial diagnosis (*r* = 0.661, *p* = 0.0004), while NLR demonstrated a correlation with lactate dehydrogenase (LDH; *r* = 0.313, *p* = 0.037). In different WHO-classified MDS groups, there are significant differences in the comparison of three groups of inflammatory markers between the groups. The MDS group with specific immunophenotypic characteristics displayed significant differences in PLR compared to the group without specific immunophenotypic characteristics. Additionally, CD34 proportion exhibited a negative correlation with NLR index (*r* = −0.296, *p* = 0.0129). And patients with low PLR index have a higher survival time than those with high PLR index (*p* = 0.007).

**Conclusion:** Inflammatory markers are correlated with the clinical characteristics of MDS, providing not only auxiliary evidence for the clinical diagnosis of MDS but also showing differences in inflammatory markers based on different tumor burdens in MDS. This serves as a reference for the treatment strategy of inhibiting the progression of MDS to acute leukemia by reversing the inflammatory trend.

## 1. Introduction

Myelodysplastic syndrome (MDS) is a heterogeneous group of stem cell disorders characterized by ineffective hematopoiesis, resulting in decreased peripheral blood cells due to differences between blood and hematopoietic factors. The transformation to acute myeloid leukemia (AML) is a possible consequence [1]. Studies have shown that inflammation can lead to selective growth of abnormal stem cells, promoting tumor cell proliferation, angiogenesis, immune evasion, invasion, and migration, while also inhibiting the production of healthy blood, forming a tumor microenvironment [2]. Previous research has reported that neutrophil-to-lymphocyte ratio (NLR), platelet-to-lymphocyte ratio (PLR), and monocyte-to-lymphocyte ratio (MLR) in peripheral blood indices can reflect systemic inflammation and cancer biology, including cancer prediction, progression, and survival prognosis [3, 4]. These three inflammatory markers are relatively easy to obtain clinically, simple and practical, and are less affected by other physiological factors. However, there have been few studies attempting to explore the combined use of NLR, PLR, and MLR in relation to clinical relevance in MDS. Additionally, low blast cell percentage in MDS can easily be confused with megaloblastic anemia or aplastic anemia. Therefore, this study aims to use NLR, PLR, and MLR in combination as biomarkers for differential diagnosis of MDS, even in the early stages with low blast cell percentages. This diagnostic approach is convenient, fast, and can provide a valuable time window for patient diagnosis and treatment. We will conduct a correlation analysis of inflammatory markers in MDS with different tumor burdens and clinical characteristics, aiming to improve cytopenia and reduce the risk of leukemia progression in MDS by targeting specific inflammatory cascades.

## 2. Materials and Methods

### 2.1. Case Data

Reviewing the patient data from the hematology department of the First Affiliated Hospital of Bengbu Medical University from 2023 to 2024, we selected MDS patients who met the inclusion criteria. The selected patients underwent detailed blood routine and bone marrow examinations and diagnoses and immunophenotyping by flow cytometry. Clinical characteristics of the patients were recorded, including gender, age, peripheral blood parameters at initial diagnosis: white blood cells (WBCs), platelets (PLTs), absolute neutrophil count (ANC), absolute monocyte count (AMC), absolute lymphocyte count (ALC), lactate dehydrogenase (LDH), blast cell percentage, and blast cell immunophenotypic characteristics. Eleven patients with reduced counts in two or three blood cell lineages were selected as the control group. Among the 70 MDS patients, there were 40 males and 30 females, with a median age of 59 years. In the control group, there were five males and six females, with a median age of 51 years. This study has received ethical approval (Ethics No.: Approval No. [2023] No. 160). The clinical data of the patients are shown in Table 1.

### 2.2. Calculation of Inflammatory Index Ratios Based on Peripheral Blood Cell Counts, the Formulas for Calculation Are as Follows

  
$$\begin{array}{c} \text{NLR}=\text{ANC/ALC} \end{array}$$

  
$$\begin{array}{c} \text{PLR}=\text{platelet count/ALC} \end{array}$$

  
$$\begin{array}{c} \text{MLR}=\text{AMC/ALC}. \end{array}$$

### 2.3. Flow Cytometric Examination

**Figure 1:**
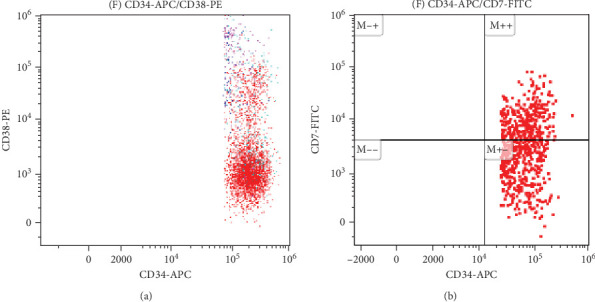
Specific immunophenotypes of MDS patients. (A) Loss of CD38 expression in original cells and (B) CD7 expression in original cells.

All antibodies were purchased from Beckman Coulter in the United States. Approximately, 20–50 μL of anticoagulated bone marrow blood is taken for cell counting. The volume of each sample is adjusted according to the cell count standard to achieve a concentration of 10^6^ cells/mL in each tube. The sample is then lysed with a red cell lysis solution, left to stand in the dark at room temperature for 10 min until the solution becomes semi-transparent. The supernatant is removed after centrifugation, and 20 μL of a mixture of monoclonal antibodies is added. After incubating in the dark for 20 min, the sample is washed with a buffer solution (PBS) and subsequently analyzed using a flow cytometer. In some MDS patients, the CD34+ primitive cells may show abnormal expression of CD7+ or lack expression of CD38, presenting as negative, as shown in Figure 1.

#### 2.3.1. Morphological Analysis

**Figure 2:**
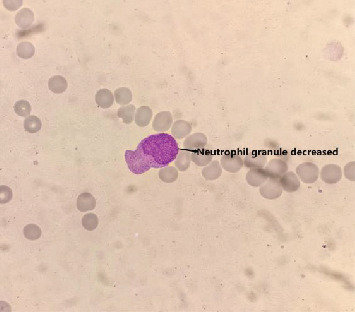
The intracytoplasmic particles of neutrophils decreased in MDS patients.

Bone marrow cytology testing for patients with MDS, the bone marrow smears need to be collected from the posterior iliac crest or anterior iliac crest. These smears will undergo Wright–Giemsa staining. Cell counting of nucleated cells should be performed at the junction between the body and tail, with a minimum count of 500 cells. In MDS patients, a reduction in granules in neutrophils can be observed (as shown in Figure 2).

### 2.4. Statistical Analysis

Data processing were conducted using SPSS 22.0 software, with ANOVA (*F*-test) for comparing multiple groups. Pearson's correlation coefficient was used to test correlations. XY scatter plots and heatmaps for multiple linear analyses were generated using Prism 8.0 software. A significance level of *p* < 0.05 indicates statistical significance of the differences.

## 3. Results

### 3.1. In the Initial Diagnosis of MDS Patients, Inflammatory Markers Were Analyzed Using ROC Curve Analysis Compared With the Control Group

**Figure 3:**
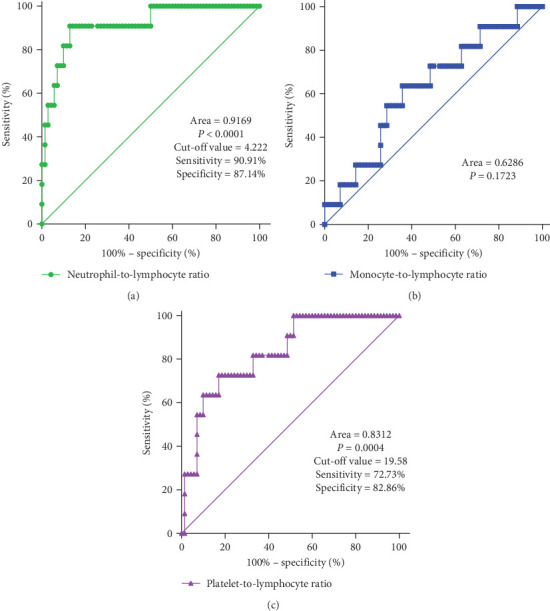
Analysis of inflammation index of MDS patients and ROC curve of control group at first diagnosis. (A) NLR, (B) MLR, and (C) PLR.

In clinical practice, it can be challenging to differentiate MDS with low blast counts. Therefore, patients with cytopenias in two or more cell lineages (granulocytes, red blood cells, and PLTs) but without MDS were selected as the control group. ROC curve analysis of inflammatory marker characteristics in MDS patients was conducted to assess the diagnostic value of these markers in identifying MDS. It is notable that the NLR levels displayed good discriminative ability between MDS patients and controls, with an area under the curve (AUC) of 0.9169 (*p* < 0.0001). Using the maximum value of the Youden index as the critical point, the cutoff value is 4.222. Additionally, PLR levels also showed discriminative ability between MDS patients and controls, with an AUC of 0.8312 (*p* = 0.0004). The cutoff value is 19.58. However, the MLR index did not show significant differences (AUC = 0.6286, *p* = 0.1723; Figure 3).

### 3.2. Correlation Analysis Between Inflammatory Markers and Clinical Parameters in MDS Patients After Conducting Multiple Linear Analyses

**Figure 4:**
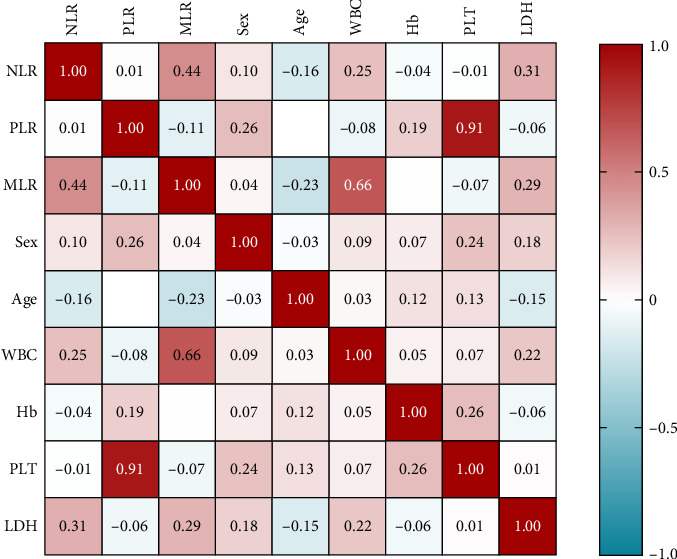
Correlation analysis of inflammation index and clinical index in MDS patients.

On clinical parameters of MDS patients, we found that MLR has a strong correlation with WBCs (*r* = 0.661, *p* = 0.0004), and NLR is correlated with LDH (*r* = 0.313, *p* = 0.037). The correlation between PLR and clinical parameters was not significant (Figure 4).

### 3.3. Differences in Inflammation Markers Between Different MDS Subtypes

**Figure 5:**
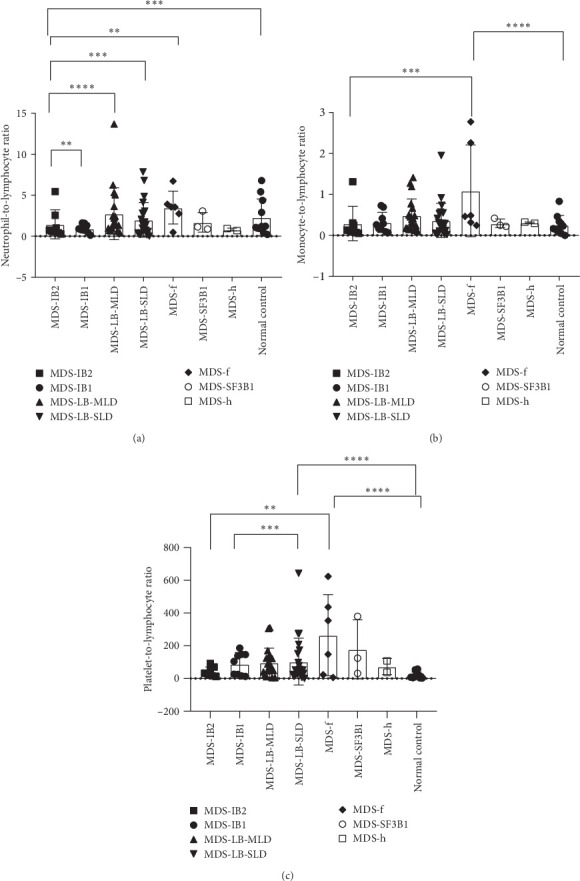
Differences in inflammation markers between different MDS subtypes: (A) NLR, (B) MLR, and (C) PLR. *⁣*^*∗∗*^*p* < 0.01; *⁣*^*∗∗∗*^*p* < 0.001; *⁣*^*∗∗∗∗*^*p* < 0.0001.

According to the 2022 WHO classification, the 70 MDS patients in this study can be divided into seven types. Comparing the differences in inflammatory markers among the groups, it was found that the NLR index showed significant differences between the MDS-IB2 group and the MDS-IB1 group, MDS-LB-MLD group, MDS-LB-SLD group, MDS-f group, and the normal control group (*p* = 0.0016, <0.0001, 0.0006, 0.0011, 0.0003). The MLR index also showed significant differences between the MDS-f group and the MDS-IB2 group, as well as compared to the normal control group (*p* = 0.0004, <0.0001). The PLR index also demonstrated significant differences when comparing pairs of groups, including the MDS-IB2 group versus MDS-f, MDS-LB-SLD versus MDS-IB1, MDS-LB-SLD versus the normal control group, and MDS-f versus the normal control group (*p* = 0.0024, 0.0007, <0.0001, <0.0001; Figure 5).

### 3.4. Differences in Inflammatory Markers Among MDS Patients With Neutrophil Granule Reduction

**Figure 6:**
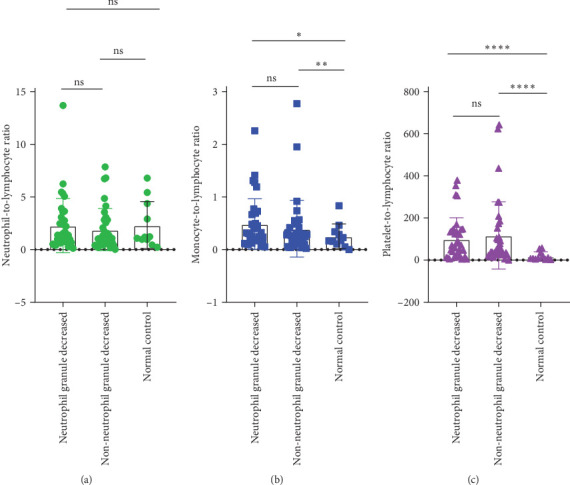
The difference of inflammatory markers in MDS group with neutropenia. (A) NLR, (B) MLR, and (C) PLR. ns: *p* > 0.05; *⁣*^*∗*^*p* < 0.05; *⁣*^*∗∗*^*p* < 0.01; *⁣*^*∗∗∗∗*^*p* < 0.0001.

In MDS patients, dysplasia of the granulocytic lineage, particularly with a reduction in granules within neutrophil cytoplasm, is a common hematopoietic abnormality. However, granule reduction can also occur during infections. To investigate this phenomenon in MDS patients, the patients were grouped based on the presence or absence of neutrophil granule reduction. In the groups with neutrophil granulocytopenia and without neutrophil granulocytopenia, there were no significant differences in NLR, MLR, and PLR. Compared to the normal control group, there are significant differences in both MLR index and PLR index between the group with reduced granules and the normal control group (*p* = 0.0189, <0.0001). Similarly, when comparing the group without granular reduction to the normal control group, there are also significant differences in MLR and PLR indices (*p* = 0.0073, <0.0001; Figure 6).

### 3.5. Differences in Inflammatory Markers Among MDS Patients With Special Immunophenotype

**Figure 7:**
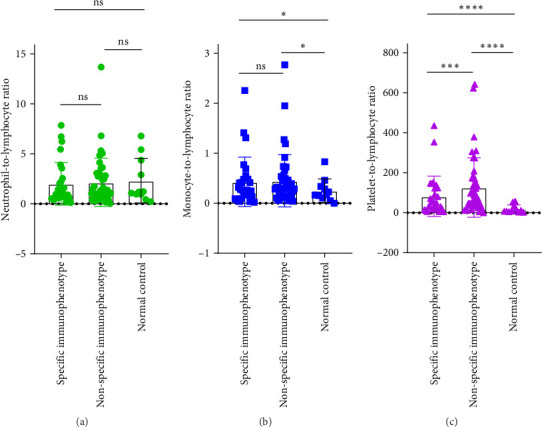
The difference of inflammatory markers in MDS group with special immunophenotype. (A) NLR, (B) MLR, and (C) PLR. ns: *p* > 0.05; *⁣*^*∗*^*p* < 0.05; *⁣*^*∗∗∗*^*p* < 0.001; *⁣*^*∗∗∗∗*^*p* < 0.0001.

Upon analyzing the immunophenotypes of MDS patients, it was found that in MDS patients with the expression of CD34+CD7+ or CD34+CD38+, compared to those without such special immunophenotypes, patients with the special immunophenotype had the following initial NLR, MLR, and PLR values: 2.004, 0.4292, and 82.57; while patients without the special immunophenotype had the following inflammatory marker values: 2.149, 0.4527, and 126.7 (*p* = 0.4761, 0.7697, 0.0392). Only the difference in PLR values was statistically significant. Comparing the special immune phenotype group with the normal control group, there are significant differences in both MLR index and PLR index (*p* = 0.0158, <0.0001). Similarly, when comparing the group without special immune phenotype with the normal control group, there are also significant differences in MLR and PLR indices (*p* = 0.009, <0.0001; Figure 7).

### 3.6. Correlation Analysis Between CD34+ Percentage and Inflammatory Markers in MDS Patients

**Figure 8:**
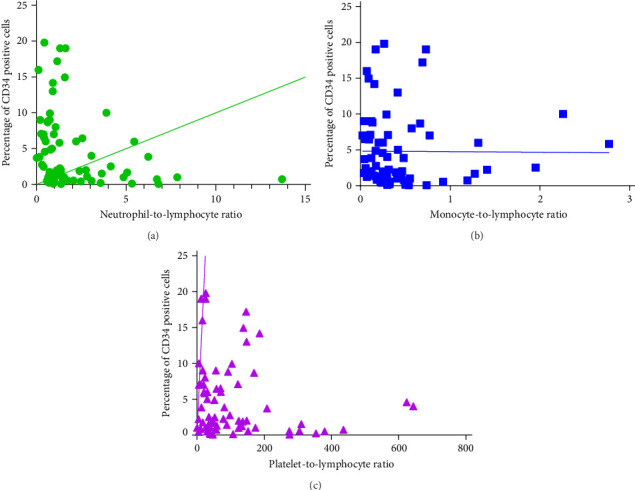
Correlation analysis of CD34+ percentage and inflammation index in MDS patients. (A) NLR, (B) MLR, and (C) PLR.

During the initial diagnosis of patients, the percentage of CD34+ cells relative to CD45+ leukocytes was measured before treatment, and correlations with NLR, MLR, and PLR were analyzed. It was found that NLR was significantly negatively correlated with the percentage of CD34+ cells, with a statistically significant difference (*r* = −0.296, *p* = 0.0129); however, there was no significant correlation between MLR and PLR with the CD34+ percentage (*p* = 0.9485, 0.1781; Figure 8).

### 3.7. Correlation Between MDS Patient Clinical Outcomes and Inflammation Markers

**Figure 9:**
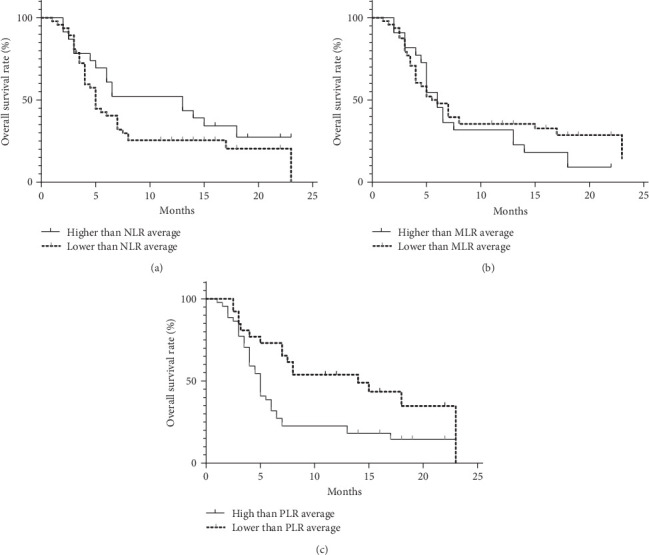
Correlation between MDS patient clinical outcomes and inflammation markers. (A) NLR, (B) MLR, and (C) PLR.

Analyzing the correlation between inflammation markers and clinical outcomes in 70 MDS patients with a 2-year follow-up period. The average values of NLR, MLR, and PLR among the 70 patients were 2.09, 0.44, and 109.06. Dividing patients into two groups based on these average values, we compared the correlation between clinical outcomes and inflammation markers. There was no significant difference in survival between the NLR and MLR groups. However, in the PLR group, the median survival time for those with values below the average was 14 months, while it was 5 months for those with values above the average. This difference was statistically significant (*p* = 0.007; Figure 9).

## 4. Discussion

In the tumor microenvironment, inflammatory mediators play a crucial role in immune surveillance and act as protective factors for patients' prognosis in malignant tumors [5]. NLR, MLR, and PLR, as inflammatory markers, have been reported to be associated with the inflammatory environment of various tumors [6, 7]. Recent studies have shown that besides genetic changes, immune dysregulation also plays a crucial role in the pathogenesis of MDS. MDS serves as a perfect model for studying age-induced information or inflammatory mechanisms, as it is a major accelerating factor in malignant cell evasion of immune surveillance by activating innate immunity [8]. The tumor microenvironment is altered by inflammation, creating a favorable environment for cancer cell proliferation [9]. Additionally, neutrophils play a key role in the development of cancer, as they produce a range of angiogenic factors affecting tumor angiogenesis. Furthermore, the cytokines and proteins contained in the granules of neutrophils promote tumor growth. These findings suggest a link between neutrophils and the progression of malignant tumors, foretelling poor prognosis. The increased expression of tumor-associated macrophages (TAMs) with an M2 phenotype in the TMEs (tumor microenvironment) is also associated with poor malignant prognosis [10]. TAMs with an M2 phenotype can promote tumor survival factors, enhance angiogenesis and tumor invasion, suppress adaptive immunity through the production of cytokines and chemokines, thereby facilitating tumor growth [11]. Monocytes in peripheral blood might indicate the development or existence of TAMs [12]. Likewise, tumor-associated neutrophils (TANs) exhibit functions that can either promote or inhibit tumor growth, akin to macrophages [13]. Cancer cells can trigger the secretion of granulocyte colony-stimulating factors, which may lead to a rise in neutrophil counts. Similarly, cancer cells can also be engulfed by activated T cells. This process leads to the proliferation of lymphocytes and significant changes in neutrophils [14–16]. Lymphocytes play a vital role in the host immune system and have anti-tumor effects. Elevated levels of lymphocytes are associated with better prognosis in various tumors [17]. In reaction to inflammation, PLTs are activated, clump together, and their quantity rises [18]. Tumor cells activate PLT indirectly via tissue factor (TF). The activation of PLT leads to tumor angiogenesis and growth through different mediators like vascular endothelial growth factor (VEGF), transforming growth factor beta (TGF-β), and platelet-derived growth factor (PDGF). Additionally, activated PLT promotes tumor cell apoptosis through cytotoxic mechanisms involving MMP and facilitates tumor invasion [13]. Therefore, PLT contributes to tumor growth, invasion, and angiogenesis [19], playing a role in tumor progression. Furthermore, proinflammatory cytokines trigger platelet activation, recruit neutrophils, and enhance the inflammatory response [20]. An increase in platelet count is considered a major indicator of poor prognosis as it raises the risk of metastasis [21].

Based on the principles mentioned above, parameters in hematology, such as neutrophils, lymphocytes, PLTs, and monocytes, are components of tumor-induced systemic inflammation in the field of hematology. The roles of the immune system and the inflammatory response are vital across various phases of cancer development, including initiation, invasion, promotion, and the formation of metastases [22]. In general, the existence of inflammation and related inflammatory markers correlates with cancer prognosis, positioning them as potential indicators for clinical prognosis [19].

Pathological Process of MDS and Chronic Inflammation Microenvironment. This study has confirmed that peripheral blood inflammation markers (NLR, PLR, MLR) are significantly abnormal in MDS patients and are associated with disease subtypes and tumor burden. ROC curve analysis shows that NLR and PLR have diagnostic significance in distinguishing MDS from nonhematopoietic system tumors (AUC = 0.9188 and 0.8317, *p* < 0.0001). Furthermore, there are differences in inflammation markers among MDS patients with different WHO classifications. Further analysis indicates that NLR is negatively correlated with the proportion of CD34+ cells (*r* = −0.3382, *p* = 0.0201), suggesting that it may indirectly reflect the degree of tumor clone expansion. This is consistent with previous studies [23, 24] where inflammation markers were found to predict cancer outcomes. Additionally, this study found that patients with lower PLR indices have a longer survival time compared to those with higher PLR indices, with a statistically significant difference (*p* = 0.007).

The proportion of primitive cells and different immune phenotypes in MDS also affects the distribution of lymphocytes related to immune function and activated T cells. As MDS progresses, primitive cells tend to shift from relatively mature to more immature stages. For example, CD117 and CD7 are more commonly found in high-risk MDS, while CD10 and CD15 are more common in low-risk MDS. CD7, as a pan-T cell antigen, can be abnormally expressed across lineages in AML, and patients with CD7+ AML have a shorter survival period than CD7− patients [25]. Studies have shown that high expression of CD7 indicates a poor prognosis in MDS patients, and abnormal expression of CD7 on CD34+ cells in MDS is associated with transformation to AML [26]. In MDS patients, there is a subset of CD34+CD38 dim/− primitive cells, which are very early primitive cells. Compared to CD38+ primitive cells, CD38 dim/− primitive cells have greater differentiation potential, lower reactivity to growth factor stimulation, and have long been considered as leukemia stem cells (LSCs) associated with leukemia [27]. This study further found that MDS patients with CD7+ or CD34+CD38 dim/− phenotype exhibit a significantly reduced PLR, suggesting an interaction between abnormal immune phenotype and inflammatory response, which may be related to the immune escape mechanism of their LSCs.

In MDS patients, the myeloid, erythroid, and megakaryocytic lineages often exhibit dysplastic hematopoiesis. Neutrophils may show a decrease or absence of granules, which mainly consist of azurophilic granules and specific granules. These granules play a crucial role in neutrophils' ability to kill and digest microorganisms. Observing the presence of granules in neutrophils is significant in determining an individual's infection status. The absence of granules in neutrophils in MDS patients may be due to infections or the action of tumor factors. In this study, comparing MDS with reduced or absent granules in myeloid morphology with MDS with normal granulocyte morphology revealed no significant differences.

In recent years, the study of tumor immune microenvironment has been increasingly emphasized. People have realized that besides eliminating tumor cells, improving the tumor microenvironment plays a crucial role in the persistence of treatment effects. Among the various components of the tumor microenvironment, the inflammatory environment is one of the most important. Therefore, investigating the relationship between the inflammatory environment and tumor occurrence and development, especially in the early stages of cancer, is paramount. Previous studies have shown that the correlation between MDS and inflammation is primarily reflected in the expression levels of inflammatory factors in MDS patients [28]. The concentrations of TNF-α, IL-6, and IL-8 in the peripheral blood and bone marrow of MDS patients are significantly elevated compared to healthy controls. The study on the differential expression and regulatory mechanisms of Th22 cells and Th17 cells in MDS patients has also revealed the role of inflammatory immune response in the pathogenesis of MDS [29]. This further confirms the abnormal expression of these inflammatory factors in MDS and their possible involvement in the immune pathogenesis of MDS. The core pathological mechanism of MDS is not only clonal hematopoietic abnormalities, but also immune microenvironmental imbalance. A chronic inflammatory state can promote the expansion of malignant clones. A persistent inflammatory microenvironment can induce DNA damage, promote genetic mutations, and accelerate the expansion of malignant clones.

In low-resource clinical settings, NLR and PLR can be used as auxiliary tools for MDS screening due to their convenience in detection. This study found that NLR is significantly correlated with LDH levels (*r* = 0.313, *p* = 0.037), and MLR is significantly correlated with WBC count (*r* = 0.661, *p* = 0.0004), supporting their use in monitoring disease activity. However, the nonspecificity of inflammatory markers requires that they be interpreted in combination with morphology, molecular markers, and IPSS-R scores. Additionally, the correlation between platelet elevation and risk of metastasis, the association between increased PLR index and shortened OS, and the positive regulation of proinflammatory cytokines on platelet activation all suggest that targeting inflammatory pathways may become a new direction for MDS treatment. There is a complex bidirectional relationship between MDS and inflammatory indicators. The chronic inflammatory microenvironment is both a driving factor and a pathological consequence of MDS. Abnormal elevations of inflammatory indicators not only reflect disease activity but also have a certain correlation with disease prognosis.

## 5. The Limitations of This Study

To minimize potential biases in our research, we have taken careful measures to ensure a high level of homogeneity in our case selection process, thereby excluding cases that presented with severe complications or coexistence of other diseases. But the classification framework of this study may not fully reflect the biological heterogeneity of MDS, especially the differences in the molecular mechanisms of abnormal development of different cell lines. Future studies can resolve the specific effects of hemocytic abnormalities on treatment response at a higher resolution level through techniques such as single-cell sequencing. As our study is conducted in a single-center setting, the conclusions drawn may be susceptible to the influences of specific medical environments and the characteristics of the patient populations involved. Therefore, to truly verify the generalizability of our results, it is essential to replicate the study in different medical institutions and among diverse patient populations.

## 6. Conclusion

Inflammation indicators play an important role in early screening, risk stratification, subtype differentiation, and treatment monitoring of MDS, especially as an effective complement to molecular techniques when molecular testing is limited. This study systematically explored the multidimensional associations between inflammatory indicators, immune phenotypes, tumor burden, and clinical stratification in MDS, providing evidence for the clinical application of inflammation markers. However, further research is needed to investigate the mechanisms underlying these relationships. Future studies should aim to clarify the role of specific inflammatory pathways in different MDS subtypes for more precise stratified treatment, optimize the whole-course management of MDS patients, and explore the potential value of targeted therapy to improve outcomes.

## Figures and Tables

**Table 1:** Clinical characteristics of 70 MDS patients.

Characteristics	Data
Age (year)	59 (22–80)
Sex	
Male	40 (57.1%)
Female	30 (42.9%)
WHO classification	
MDS-LB-SLD	23 (32.9%)
MDS-LB-MLD	19 (27.1%)
MDS-IB-1	8 (11.4%)
MDS-IB-2	9 (12.9%)
MDS-f	6 (8.6%)
MDS-SF3B1	3 (4.3%)
MDS-h	2 (2.9%)
International Prognostic Scoring System Revised (IPSS-R)	
Very low	24 (34.3%)
Low	22 (31.4%)
Intermediate	19 (27.1%)
High	5 (7.1%)

## Data Availability

The data that support the findings of this study are available on request from the corresponding author. The data are not publicly available due to privacy or ethical restrictions.
